# Development of a Musculoskeletal Ultrasound Protocol to Evaluate Hand Pain in Systemic Sclerosis Patients

**DOI:** 10.3390/diagnostics14070669

**Published:** 2024-03-22

**Authors:** Meridith L. Balbach, Robert Corty, Bradford Hill, Tracy Frech, Fawad Aslam, Erin Y. Chew

**Affiliations:** 1 Division of Rheumatology and Immunology, Vanderbilt University Medical Center, Nashville, TN 37232, USA; meridith.balbach@vumc.org (M.L.B.); robert.corty@vumc.org (R.C.); tracy.frech@vumc.org (T.F.); 2 Department of Plastic Surgery, Vanderbilt University Medical Center, Nashville, TN 37232, USA; brad.hill@vumc.org; 3 Mayo Clinic in Arizona, Department of Rheumatology, Scottsdale, AZ 85259, USA

**Keywords:** musculoskeletal ultrasound, systemic sclerosis, hand pain

## Abstract

Hand impairment is a frequently reported complaint in systemic sclerosis (SSc) patients and a leading cause of disability and diminished quality of life. Managing hand pain can be particularly challenging due to the coexistence of non-inflammatory arthralgias, inflammatory arthritis, acro-osteolysis, tenosynovitis, joint contractures, tendon friction rubs, nerve entrapment, Raynaud’s phenomenon (RP), digital ulcers (DU), sclerodactyly, calcinosis, and chronic pain. While physical examination and radiographs are the first line methods for evaluating hand pain, they are limited in scope and miss many underlying etiologies of hand impairment. We propose a joint ultrasound (US) hand protocol to differentiate between various articular, periarticular, ischemic, skin, and nerve pathologies and to assist in targeted treatment strategies.

## 1. Introduction

Systemic Sclerosis (SSc) is a rare multiorgan-system rheumatic condition characterized by vasculopathy, fibrosis, and autoimmunity. Hand impairment is a frequently reported complaint in systemic sclerosis (SSc) patients and a leading cause of disability and diminished quality of life. Managing hand pain can be particularly challenging due to the coexistence of non-inflammatory arthralgias, inflammatory arthritis, acro-osteolysis, tenosynovitis, joint contractures, tendon friction rubs, nerve entrapment, Raynaud’s phenomenon (RP), digital ulcers (DU), sclerodactyly, calcinosis, and chronic pain. While physical examination and radiographs are the first line methods for evaluating hand pain, they are limited in scope and miss many underlying etiologies of hand impairment.

Musculoskeletal ultrasound (MSUS) is rapidly becoming a mainstay diagnostic tool in the assessment of rheumatic diseases given its low cost, portability, and safety as a non-ionizing imaging modality. The Outcome Measures in Rheumatology (OMERACT) Ultrasound (US) working group has provided definitions for pathologic lesions seen in various rheumatic disorders, specifically in inflammatory arthritis, offering a valuable framework for the assessment of hand impairment in systemic sclerosis [1,2]. MSUS is more sensitive than combined joint exams and hand radiographs and can be used to provide a comprehensive assessment of the broad spectrum of hand impairment in SSc. The feasibility and efficiency of performing a protocolized point-of-care MSUS exam can ultimately assist in targeted treatment strategies.

However, implementation of MSUS as a part of standard care is challenging due to the need for advanced training in US to recognize hand pathologies specific to SSc patients. Additionally, time constraints for the rheumatologist may prevent point-of-care MSUS during the clinic visit. 

To our knowledge, there is currently no standardized published MSUS protocol for the evaluation of hand pain in SSc patients. In this technical report, we provide a comprehensive review of the spectrum of hand pathology in SSc as seen on US and recommend an efficient MSUS hand protocol that can be performed in less than 10 min for the detection of various etiologies of hand pain. Implementation of this protocol at our center has guided the personalized management of hand disability in SSc patients.

## 2. Spectrum of Hand Pathology in Systemic Sclerosis as Seen on Ultrasound

The spectrum of hand pathology in SSc encompasses specific joint, tendon, and nerve pathologies, as well as ischemia, skin fibrosis, and calcium deposition.

### 2.1. Joint Pathology

#### 2.1.1. Non-Inflammatory Arthralgias

**Figure 1:**
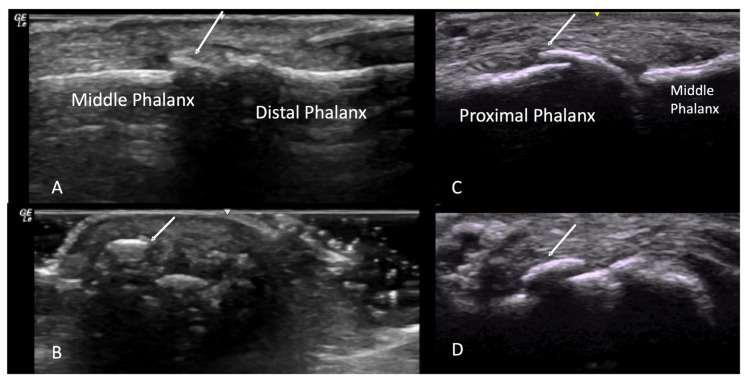
Long axis views of osteophytes (indicated by arrows) at DIP (**A**) and MCP (**C**) joint in long view. Confirmation of step-up bony deformities seen in short axis views at DIP (**B**) and MCP (**D**) joint. DIP = distal interphalangeal, MCP = metacarpal phalangeal.

Multiple studies have consistently demonstrated that SSc patients carry a higher burden of osteophytosis compared to controls. The severity of osteophytes correlates with the number of tender joints making them a substantial contributor to hand dysfunction [3,4]. Additionally, there may be an increased prevalence of erosive osteoarthritis in SSc patients [3]. On ultrasound, osteophytes are identified as step-up bony prominences at the bony margin that are visible in two perpendicular planes [1,2,5] (Figure 1).

#### 2.1.2. Inflammatory Arthritis

**Figure 2:**
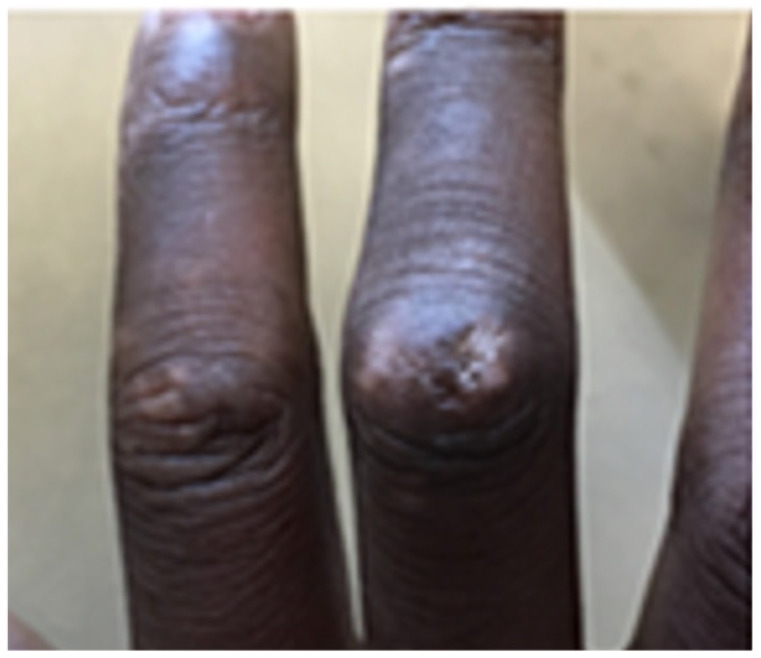
Traumatic ulcerations in systemic sclerosis patient.

In the EULAR EUSTAR registry, 16% of SSc patients exhibit synovitis [6]. Overlap erosive Rheumatoid arthritis has been reported in 1–5% of patients [7]. However, detecting true inflammatory arthritis can be very difficult. The physical examination alone poses challenges due to digital ulcerations, traumatic pits, skin tightening, and contractures, limiting the clinicians’ ability to palpate underlying synovitis in the joints and tendons [3,8] (Figure 2). Prior studies report a higher prevalence of 25–58% of patients with US-detected synovitis [3,4,9,10,11].

**Figure 3:**
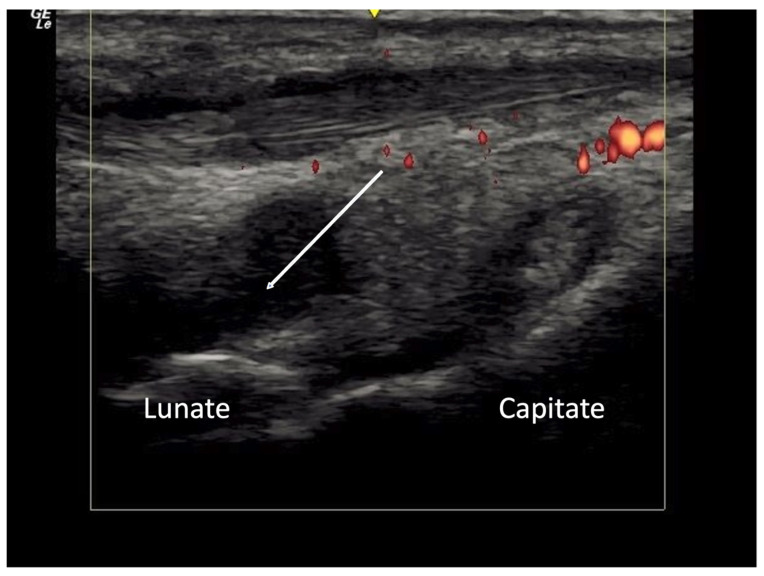
Longitudinal view of median dorsal wrist. Effusion (indicated by arrow) seen as anechoic, non-compressible material without Doppler signal within the radiocarpal joint.

**Figure 4:**
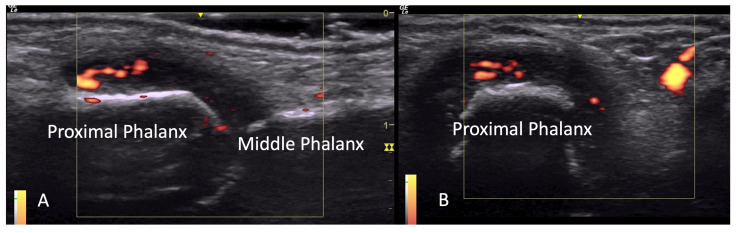
Synovitis seen as hypoechoic intraarticular material, non-compressible, with Doppler signal seen at MCP joint in longitudinal (**A**) view and transverse (**B**) view. MCP = metacarpal phalangeal.

**Figure 5:**
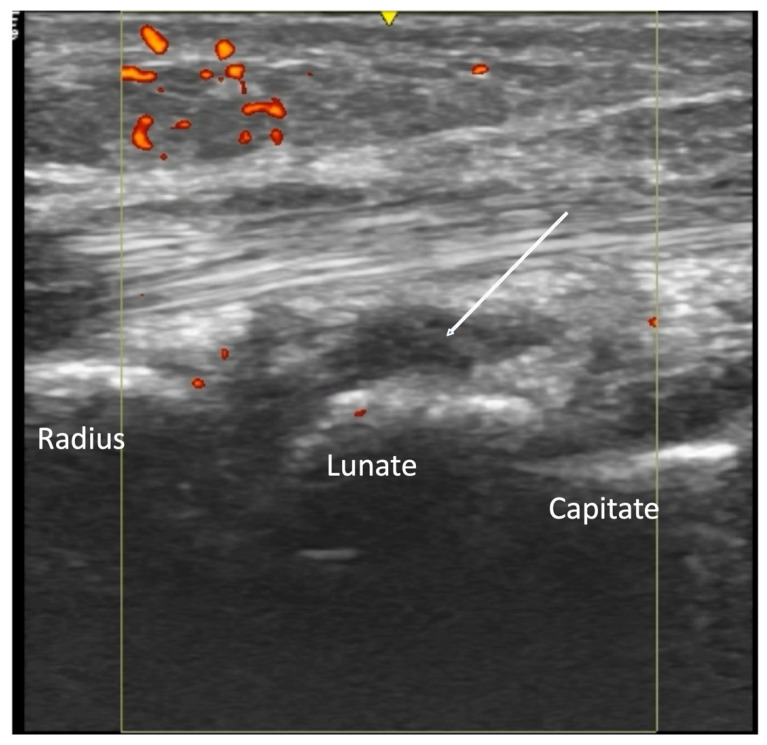
Noncompressible hypoechoic material (indicated by arrow) with no Doppler signal seen within the radiocarpal and intercarpal joint of the wrist.

**Figure 6:**
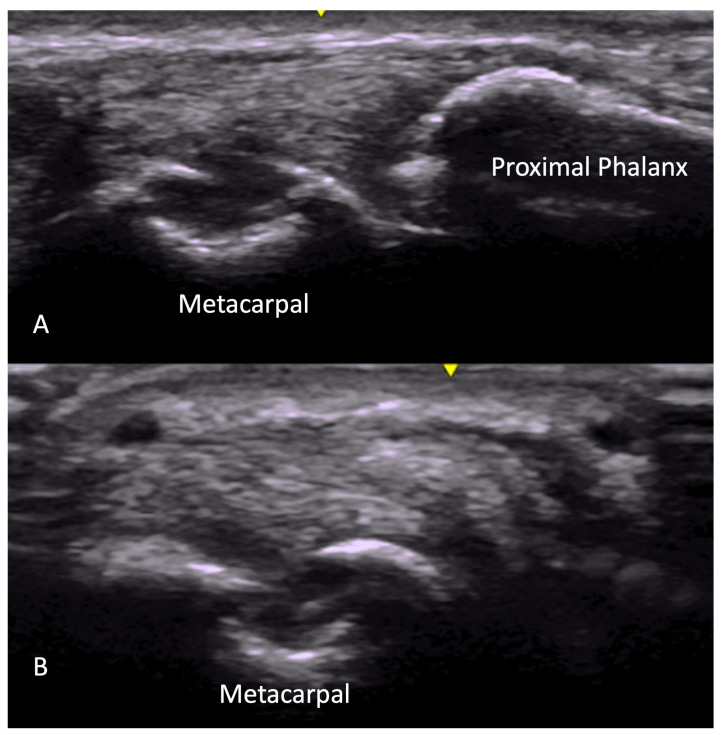
Erosion visualized as step-down deformity seen on radial view of metacarpal phalangeal (MCP) joint in long view (**A**) and short view (**B**).

Any displacement of the intra-articular triangle of fatty tissue or abnormal synovial tissue may be due to an effusion, synovial hypertrophy, or synovitis. An effusion is defined as compressible, displaceable hypoechoic or anechoic material that does not display a Doppler signal (Figure 3). According to OMERACT definitions, synovitis is defined as abnormal hypoechoic intraarticular synovial tissue that is non-displaceable and poorly compressible with or without a Doppler signal (Figure 4). However, in cases where no Doppler signal is seen, the pathology is more accurately termed as synovial hypertrophy (Figure 5) while the presence of a Doppler signal suggests hyperemia, more aptly termed synovitis. Erosions are another elementary lesion that can be seen in the inflammatory arthritis of SSc and are defined as an intra- and/or extra-articular discontinuity of the bone surface visible in two perpendicular planes [1,2,5] (Figure 6).

**Figure 7:**
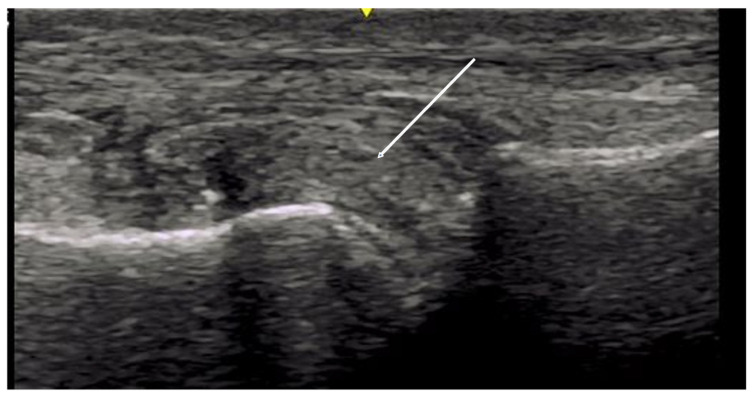
Hyperechoic, heterogenous, well-circumscribed material (indicated by arrow) seen within intraarticular space of MTP joint indicative of tophus. MTP = metatarsal phalangeal.

**Figure 8:**
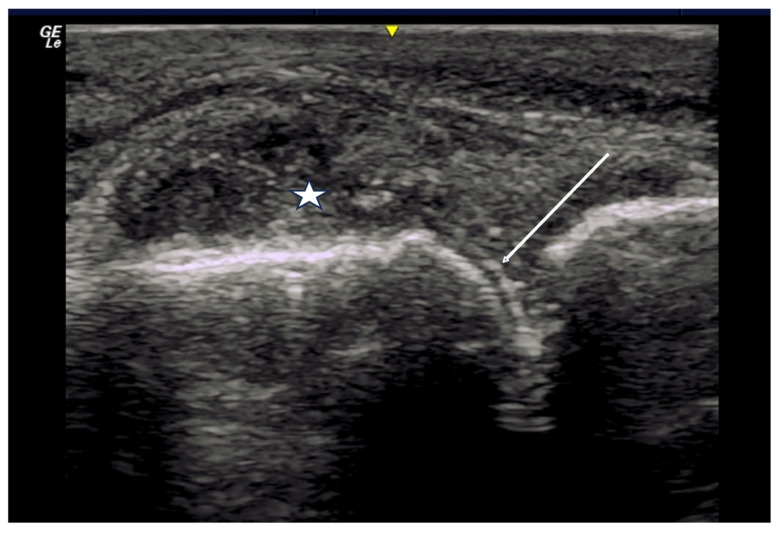
Arrow indicates double contour sign of irregular hyperechoic material layering over the surface of hyaline cartilage of the metatarsal head. Tophaceous material also visualized distending joint capsule as evidenced by star.

**Figure 9:**
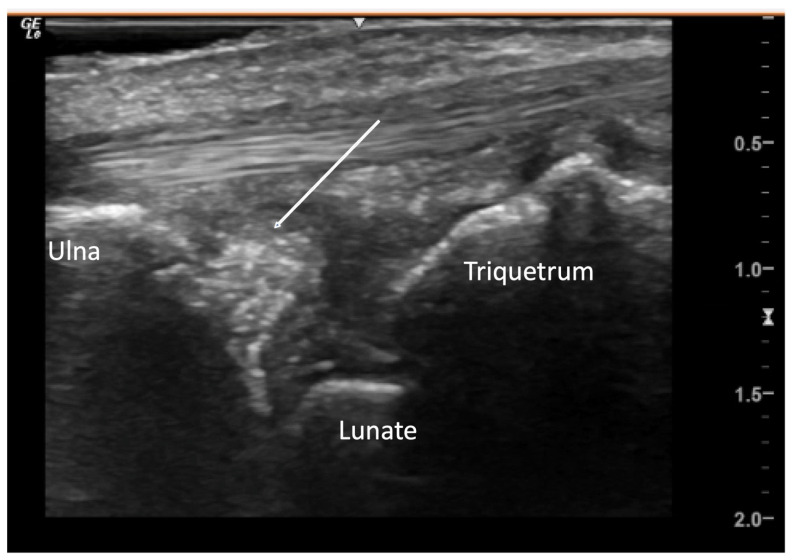
Arrow indicates hyperechoic material suggestive of calcification within the triangular fibrocartilage on medial longitudinal view of the wrist.

**Figure 10:**
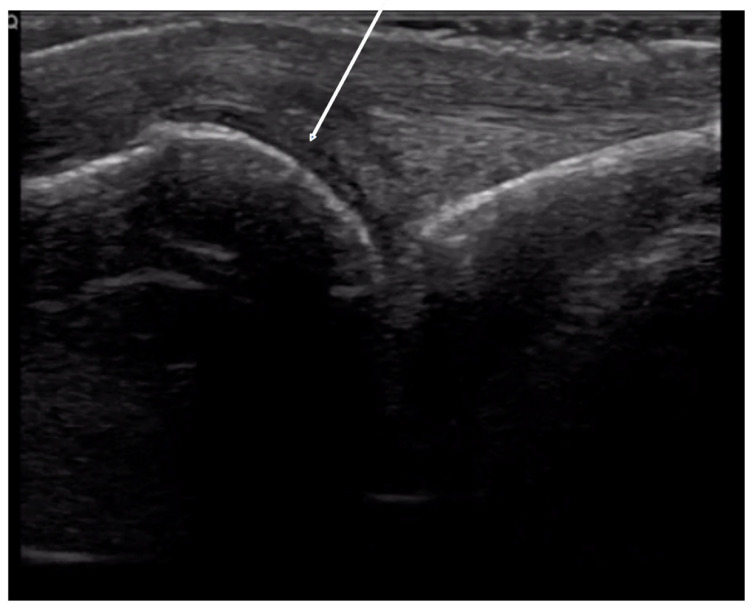
Intra-hyaline cartilage calcification (indicated by arrow) seen at MCP joint. MCP = metacarpal phalangeal.

Although lacking systematic prevalence estimates, crystalline arthritis, including gout and calcium pyrophosphate deposition (CPPD) disease, must be considered a comorbid condition in SSc. Specific ultrasound findings that can support the presence of gout include tophi, the double contour sign, and erosions. Tophi are a well-circumscribed, hyperechoic, heterogenous material with an anechoic rim, that may or may not generate posterior acoustic shadowing, depending on the degree of calcification [12] (Figure 7). A double contour sign is the ultrasonographic appearance of monosodium urate crystals visualized on the surface of hyaline cartilage [12]. This hyperechoic layer typically appears irregular, bright as bone, and remains unchanged with variation in the angle of insonation (Figure 8). The OMERACT definitions for CPPD disease include hyperechoic deposits that do not create posterior shadowing within fibrocartilaginous structures (Figure 9), the hyaline cartilage (Figure 10), tendons, or synovial fluid [13,14].

#### 2.1.3. Acro-Osteolysis

**Figure 11:**
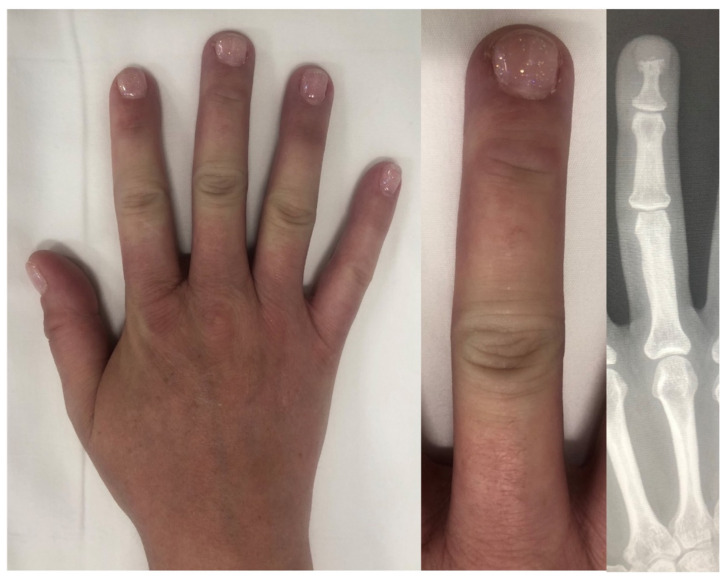
Acro-osteolysis detected on radiographs but not visually seen on physical exam.

**Figure 12:**
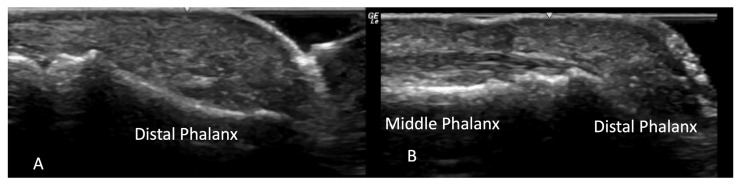
Normal concave bone contour of distal phalanx on volar view (**A**). Evidence of acro-osteolysis with disappearance of concave cortical outline of the distal phalanx (**B**).

Acro-osteolysis, distal phalanx resorption, occurs in about 25% of SSc patients and is thought to be related to repeated ischemic insult and retractile pressure from skin thickening [7]. Notably, acro-osteolysis can occur without clear physical exam findings, highlighting the value of ancillary studies for early detection (Figure 11). Severe cases can lead to finger foreshortening, causing significant cosmetic distress for patients. On ultrasound, acro-osteolysis is defined as the disappearance of the concave cortical outline of the distal phalanx or the abrupt ending of the dorsal cortex [15] (Figure 12).

### 2.2. Tendon Pathology

#### 2.2.1. Tenosynovitis

**Figure 13:**
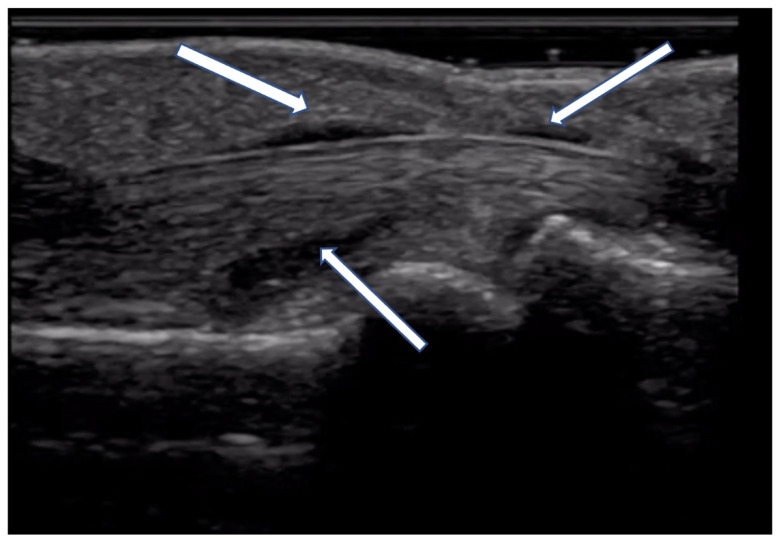
Tenosynovial effusion (indicated by arrow) seen with compressible anechoic material distending flexor tendon sheath.

**Figure 14:**
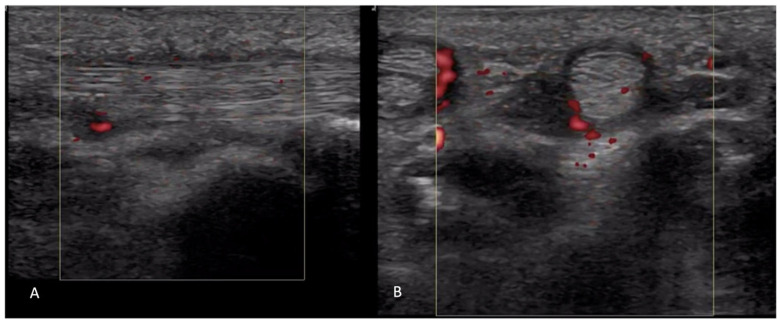
Tenosynovitis of flexor tendon seen in long axis (**A**) and short axis (**B**).

Prior studies have reported a prevalence of US-detected tenosynovitis in 27–65% of SSc patients [15,16]. OMERACT defines tenosynovitis as abnormal anechoic and/or hypoechoic (relative to tendon fibers) tendon sheath widening which can be related to both the presence of tenosynovial abnormal fluid or hypertrophy [1,2] (Figure 13). Ultrasound helps further classify tenosynovitis into inflammatory or sclerosing patterns. Inflammatory tenosynovitis is associated with a power Doppler signal (Figure 14), while sclerosing tenosynovitis is characterized by hyperechoic tendon sheath thickening [10]. Notably, tenosynovitis is more commonly seen in extensor tendons compared to flexor tendons, with the sclerosing pattern being more prevalent than the inflammatory pattern [16]. 

#### 2.2.2. Contractures and Tendon Friction Rubs

**Figure 15:**
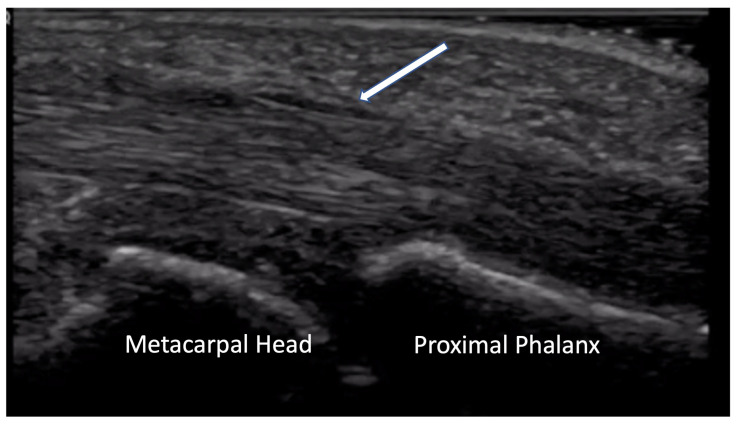
Thickening of A1 pulley (indicated by arrow) superficial to flexor tendon at level of MCP joint. MCP = metacarpal phalangeal.

Joint contractures, particularly fixed flexion contractures of the proximal interphalangeal (PIP) joint, are a leading cause of disability and social discomfort for patients, affecting approximately 31% of patients [6]. Finger flexion contractures are postulated to result from abnormalities in the flexor–tendon complex, including a thickening of the A1 pulley, along with peritendinous and soft tissue calcifications [17,18]. Ultrasound assessments demonstrate that A1 pulley thickness positively correlates with disease duration and negatively correlates with hand mobility [19] (Figure 15). 

Tendon friction rubs, detected as coarse crepitus with joint movement, are thought to be due to fibrin deposition on tendon sheaths and overlying fascia [20]. These rubs are observed in about 11% of patients in the EUSTAR registry and are associated with increased risks for digital ulcers, muscle weakness, pulmonary fibrosis, and renal involvement [6,21]. Ultrasound findings associated with tendon friction rubs include a thickening of the A1 pulley, and a thickening of the retinacula and extensor/flexor tendons, as well as tenosynovitis [4,18]. 

### 2.3. Nerve Pathology

#### Carpal Tunnel Syndrome

**Figure 16:**
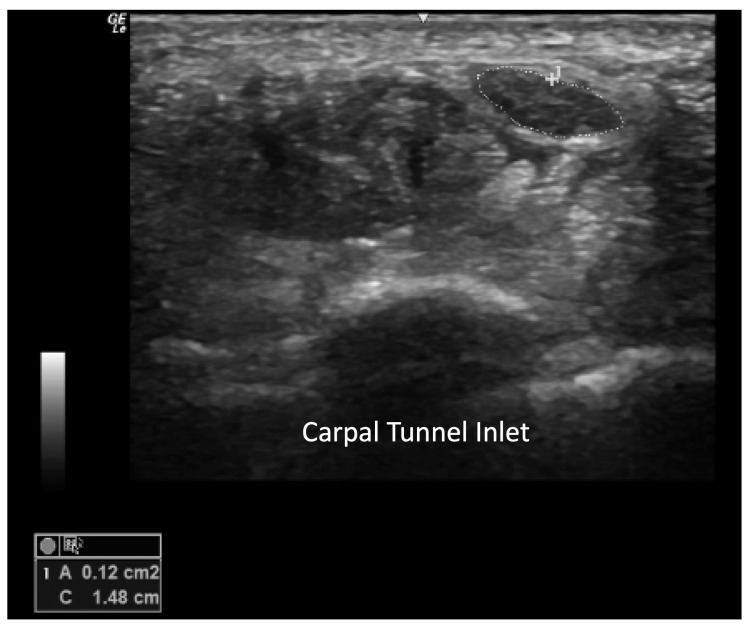
Cross sectional area of median nerve at the level of the carpal tunnel inlet measuring greater than 0.12 cm^2^ has a positive likelihood ratio of 19.9 for carpal tunnel syndrome.

The swelling of tendon sheaths and synovitis in the vicinity of the carpal tunnel inlet can lead to the development of carpal tunnel syndrome. Few studies support an increased risk for median nerve entrapment in SSc patients compared to healthy controls, though the exact prevalence is unclear. In a systematic review investigating the prevalence of peripheral neuropathy in SSc, a compression neuropathy was reported in 26% of studies with median nerve entrapment being the most common form [22]. The median nerve cross-sectional area can be measured for diagnosing impingement [23]. A median nerve size of less than 8 mm^2^ yields a negative likelihood ratio for CTS of 0.13, while a CSA of greater than or equal to 12 mm^2^ corresponds to a positive likelihood ratio of 19.9 [24]. An additional valuable measure involves evaluating the median nerve 12 cm proximal to the carpal tunnel inlet [24]. A wrist-to-forearm ratio of 1.4 or more showed 100% sensitivity for diagnosing CTS [25] (Figure 16). 

### 2.4. Vascular Pathology

Repeated vascular ischemia drives much of the hand pathology seen in SSc. Raynaud’s phenomenon, characterized by the classic triphasic finger discoloration (white to blue to red) affects up to 95% of SSc patients and is often their initial symptom [20]. Digital ulcers occur secondary to repeated vascular insults and can be complicated by infection, sometimes requiring subsequent amputation. In the ECLIPSE study, digital ulcers were significantly associated with pain and disability [26]. 

**Figure 17:**
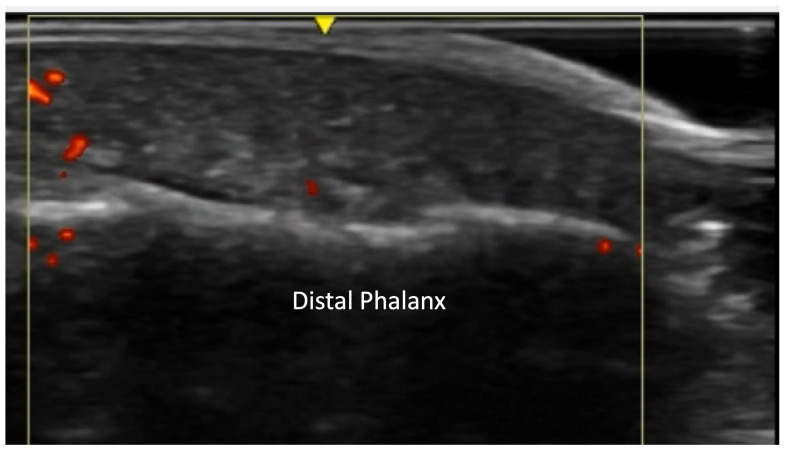
Absence of finger pulp flow in the distal phalanx in a systemic sclerosis patient.

Ulnar artery occlusion and finger pulp blood flow are two imaging biomarkers associated with new or recurrent digital ulcers [27,28,29]. Although OMERACT lacks consensus definitions for these two sonographic findings, studies have detailed techniques to standardize these measurements. Ulnar artery blood flow occlusion is defined as the complete cessation of blood flow by the power Doppler signal [8,28]. The loss of finger pulp blood flow is defined as the absence of the power Doppler signal in the sub hypodermal finger pulp in at least one of the two evaluated fingers per hand [8] (Figure 17).

### 2.5. Skin Thickening

Puffy hands are thought to be a consequence of edema preceding fibrotic changes in the skin, making them one of the first non-RP symptoms seen in SSc [20]. As the disease progresses, sclerodactyly becomes a leading cause of hand disability, increasing the risk for small joint contractures and digital ulcers. While the use of US to assess skin thickness is not widely adopted into clinical practice, there are numerous studies suggesting a positive correlation between US-measured skin thickness and histological skin thickness [30,31].

### 2.6. Calcium Deposition

Calcinosis cutis, the deposition of calcium in the skin and subcutaneous tissues, affects about 22% of patients and is thought to result from underlying chronic ischemia [32,33]. These calcium deposits often occur at trauma-prone sites on the volar surface of fingertips [26,27]. The local inflammation caused by calcium deposits can lead to skin ulceration and infection, ultimately compromising hand function and diminishing overall quality of life [7,32,34]. 

**Figure 18:**
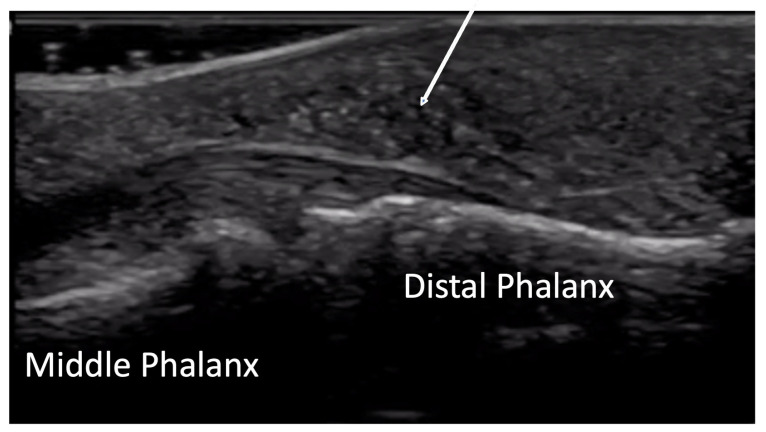
Hyperechoic well-circumscribed lesions (indicated by arrow) without shadowing located in the skin and subcutaneous soft tissue in a systemic sclerosis patient.

US can detect calcinosis with high sensitivity (89%) compared to radiographs [15]. These calcium deposits are identified as hyperechoic lesions with or without shadowing located in the skin, soft tissue, tendons, peritendinous or periarticular areas [8] (Figure 18). Supporting the hypothesis that calcinosis is driven by ischemic insults, US-detected ulnar artery occlusion is found to be associated with x-ray identified calcinosis [8]. 

## 3. Materials and Equipment

A focused US examination of the hand and wrist may be targeted at the most symptomatic site but adopting a systematic approach is essential to comprehensively evaluate the spectrum of pathology observed in SSc.

The patient should sit or lie comfortably with their hand, wrist, and forearm on a supported surface between the sonographer and the ultrasound screen. A high-frequency linear probe of at least 12 MHz should be used to evaluate the superficial structures of the hand and wrist. Creating a 1–3 mm layer of gel between the skin and the transducer can help the sonographer “float the probe” over each structure, avoiding compression that may obscure pathology that may be present. 

## 4. Detailed Procedure and Expected Results: MSK US Protocol for Hand Pain in Systemic Sclerosis Patient

### 4.1. Evaluation of the Fingers

#### 4.1.1. Dorsal Aspect

**Figure 19:**
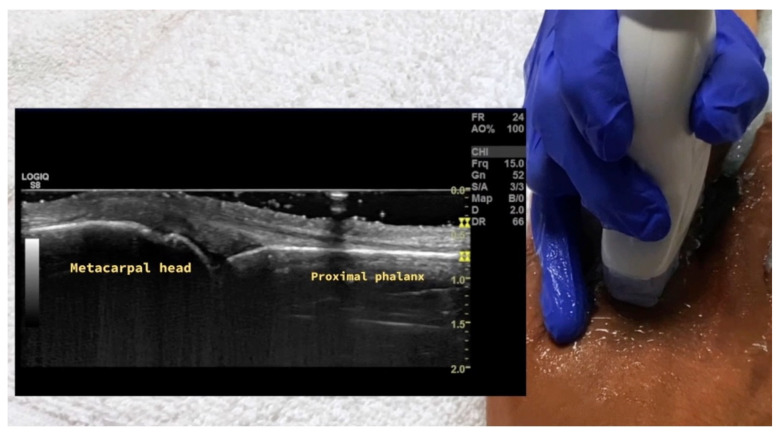
Probe placement and ultrasound of dorsal longitudinal view of MCP joint. MCP = metacarpal phalangeal.

In a typical dorsal longitudinal view of the metacarpophalangeal (MCP) joints, there is a naturally indented dip in the contour of the metacarpal head, a smooth bone contour, anechoic hyaline cartilage at the metacarpal head, a homogeneous iso-echoic intra-articular triangle of fatty tissue sitting within the joint, and a slender fibrillar extensor tendon running superficial to the joint capsule (Figure 19). This view is particularly helpful to evaluate for osteophytes characteristic of non-inflammatory osteoarthritis or erosive osteoarthritis accompanying reactive synovial hypertrophy. This view can also reveal erosions, synovitis, and tenosynovitis in cases of inflammatory arthritis. Periarticular erosions, the double contour sign, tophi, and calcification of capsular ligamentous structures can also be visualized and suggest an underlying crystalline arthritis. On dorsal longitudinal views of the distal interphalangeal (DIP) joint, deviations from the normally straight or slightly concave contour of the cortex may suggest acro-osteolysis.

#### 4.1.2. Volar Aspect

**Figure 20:**
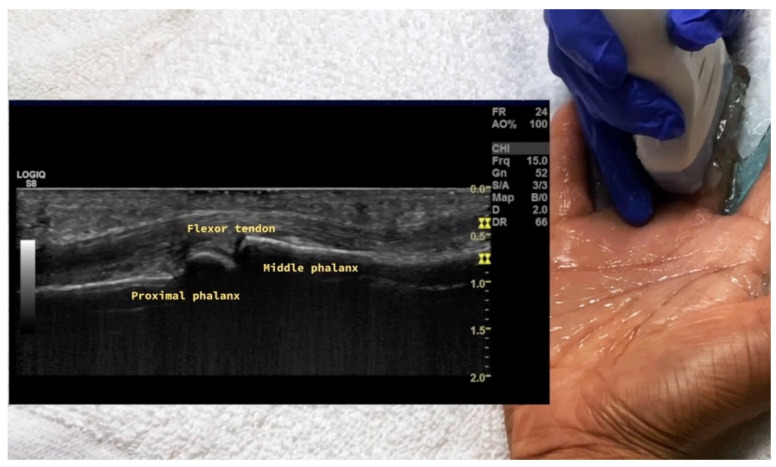
Probe placement and ultrasound of volar longitudinal view of MCP joint. MCP = metacarpal phalangeal.

Examination of the volar aspect of the MCP, PIP, and DIP joints is particularly helpful for evaluating the larger flexor tendons. Careful examination for flexor tendon tenosynovitis, the thickening of the finger pulley system, and calcifications within the tendon or in peritendinous areas should be performed (Figure 20). 

Tendons may have a thickened hypoechoic appearance indicative of a fibrotic pattern or the more classic inflammatory hypoechoic tendon sheath widening with the Doppler signal. The pulley systems are usually difficult to visualize in a normal hand. However, in patients with SSc, the pulley may exhibit diffuse hypoechoic thickening and the underlying flexor tendon may show findings of tendinosis or tenosynovitis [35]. 

Calcinosis deposits can be found in any soft tissue areas, but are commonly seen on the volar aspects of the fingertips.

#### 4.1.3. Radial and Ulnar Aspects

**Figure 21:**
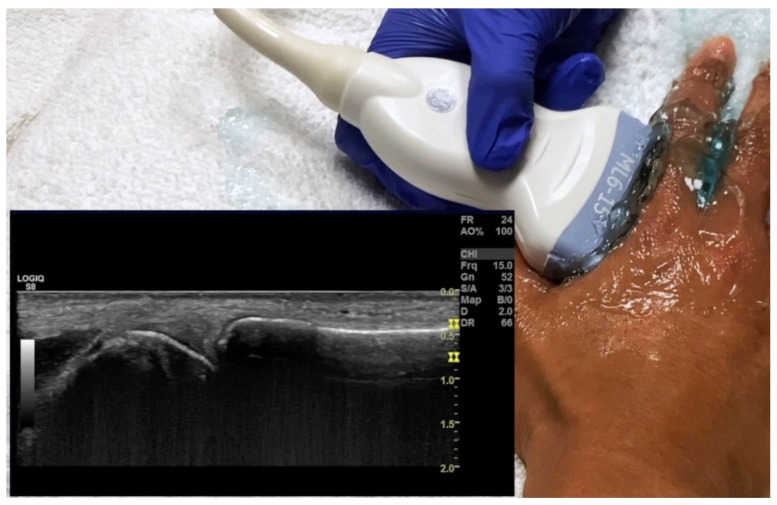
Probe placement and ultrasound of radial view of second MCP joint. MCP = metacarpal phalangeal.

The lateral aspects of each joint are helpful areas to look for erosions at sites of joint capsule insertion, particularly the ulnar view of the fifth MCP joint and the radial view of the second MCP joint (Figure 21). It is important to remember that erosions must be confirmed in orthogonal views.

### 4.2. Evaluation of the Wrist

#### 4.2.1. Dorsal Aspect

**Figure 22:**
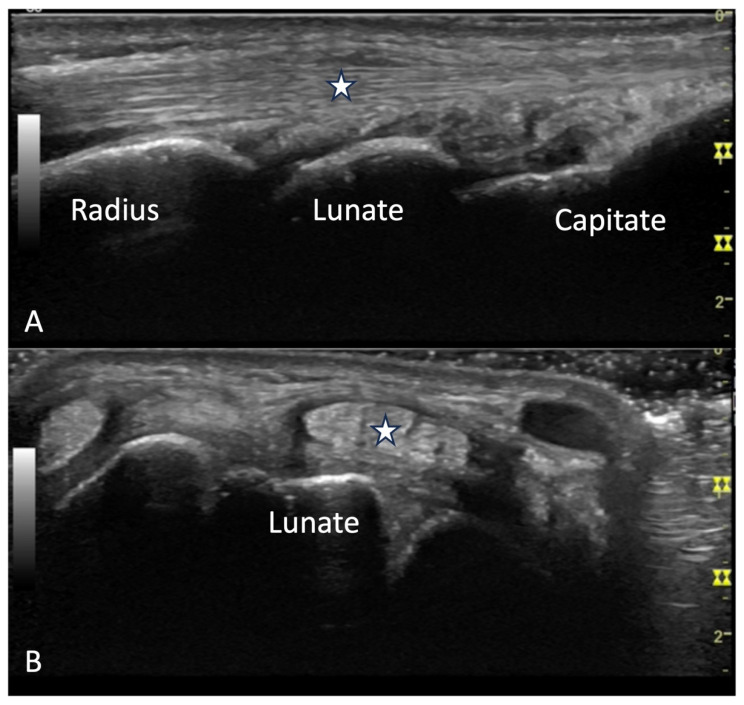
Dorsal views of wrist in long axis (**A**) and short axis (**B**). Star indicates fourth compartment extensor tendons.

Longitudinal and transverse views of the wrist are helpful to evaluate for extensor tendon tenosynovitis and the thickening of the retinaculum (Figure 22). Effusion, synovial hypertrophy, or synovitis can be visualized in the radiocarpal or intercarpal joint. The bony contour of the carpal bones should be examined for erosions or osteophytes as well.

#### 4.2.2. Volar Aspect

**Figure 23:**
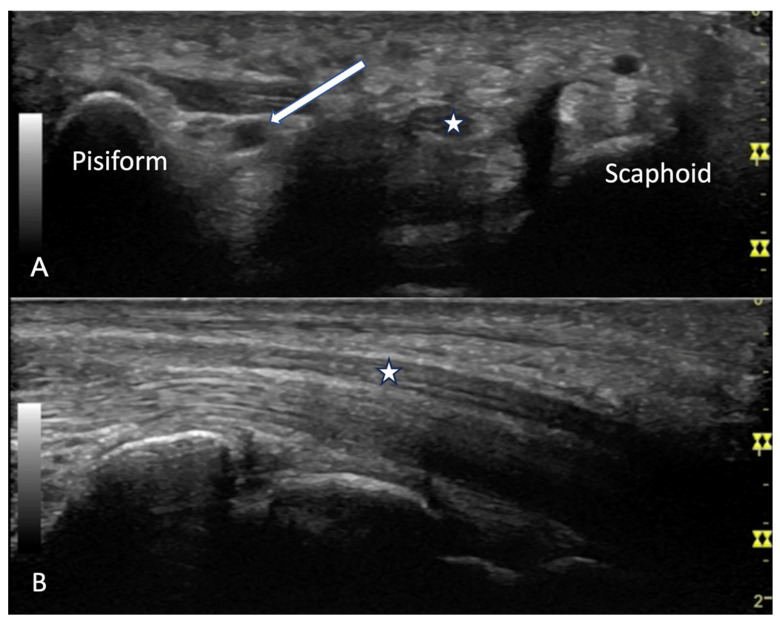
(**A**) Carpal tunnel visualized in transverse view with bony landmarks of pisiform, scaphoid. Star indicates median nerve. Flexor tendons are not visualized due to anisotropy. Arrow indicates ulnar artery. (**B**) Median volar view of wrist. Star indicates median nerve.

Wrist joint effusion, synovial hypertrophy, or synovitis can also be visualized on the volar aspect along with flexor tendon tenosynovitis. However, the volar wrist view is helpful to image the carpal tunnel and measure the cross-sectional area of the median nerve at the level of the pisiform and scaphoid in transverse view. In this view, the median nerve has a hyperechoic epineurium with a hypoechoic honeycomb appearance (Figure 23). Because the median nerve is less anisotropic, the nerve can be distinguished from the tendons when they are made maximally dark. The ulnar artery can be visualized with the Doppler mode in Guyon’s canal to evaluate for ulnar artery occlusion.

#### 4.2.3. Ulnar Aspect

**Figure 24:**
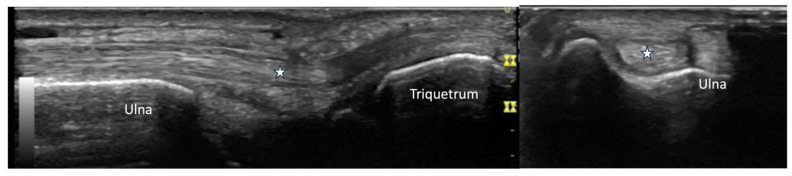
Extensor carpi ulnaris tendon (star) visualized in long axis and short axis.

The extensor carpi ulnaris (ECU) is best visualized on the ulnar aspect of the wrist and can be best seen when the wrist is slightly radially deviated (Figure 24). Deep to the ECU tendon is the triangular fibrocartilage which should be examined for calcifications that may suggest underlying crystalline arthropathy. Examination of the distal ulna for erosions is crucial, particularly in cases concerning overlap rheumatoid arthritis.

#### 4.2.4. Radial Aspect

**Figure 25:**
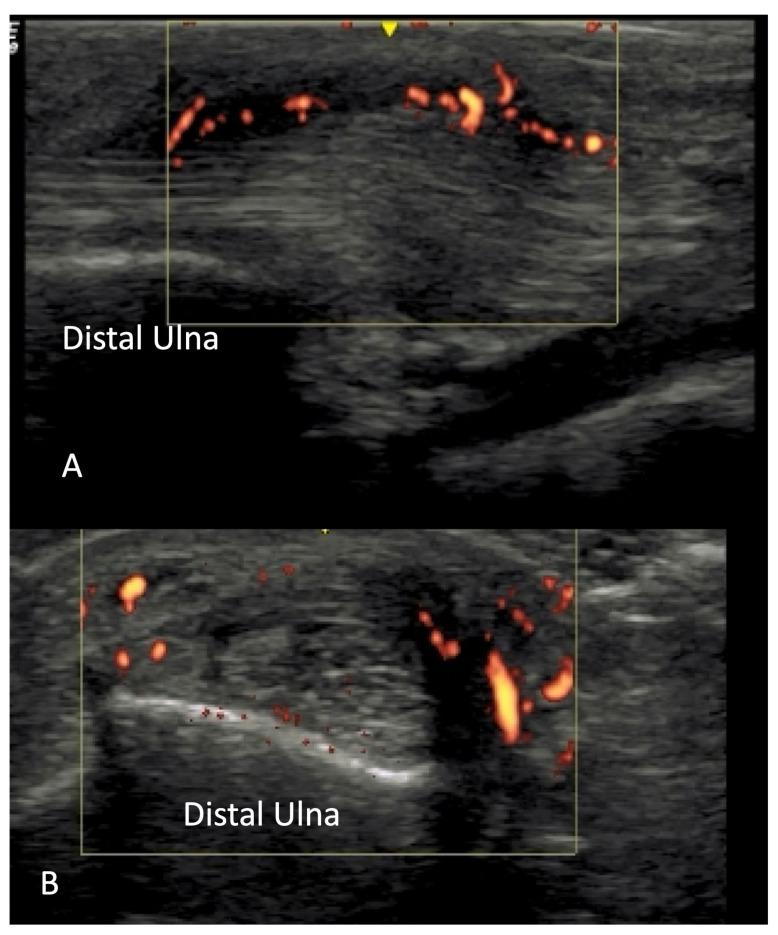
Tenosynovitis of first compartment tendons of wrist suggestive of DeQuervain’s tenosynovitis in long axis (**A**) and short axis (**B**).

Thickening of the first compartment tendons of the wrist, extensor pollicus brevis and abductor pollicus longus along with their tendon sheath and the surrounding retinaculum should be evaluated for DeQuervain’s tenosynovitis (Figure 25).

## 5. Experimental Design: Personalized Management of Hand Pain

We present preliminary data exploring the patient and provider perception of the influence of this MSUS scanning protocol on SSc disease understanding and treatment strategies at our center.

**Figure 26:**
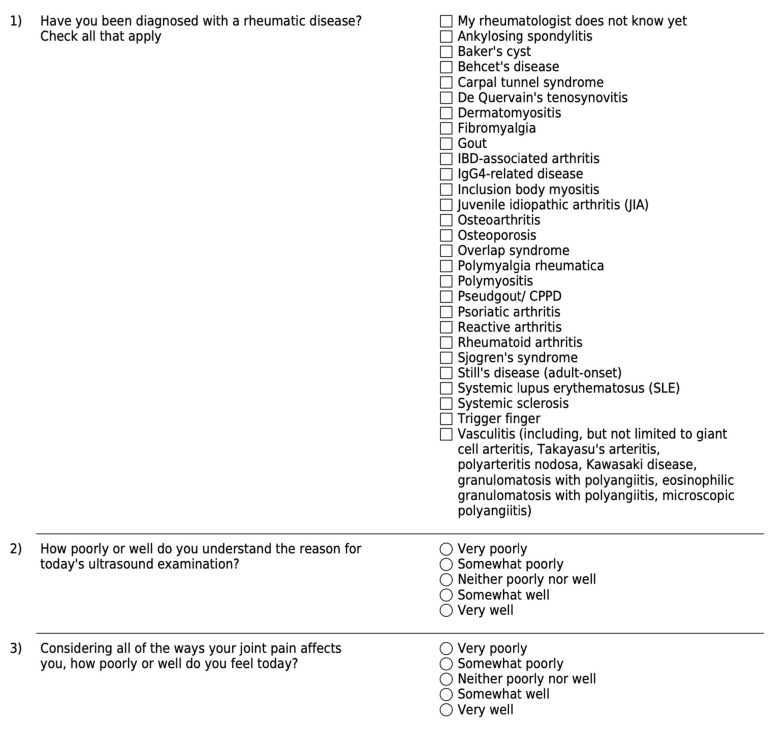

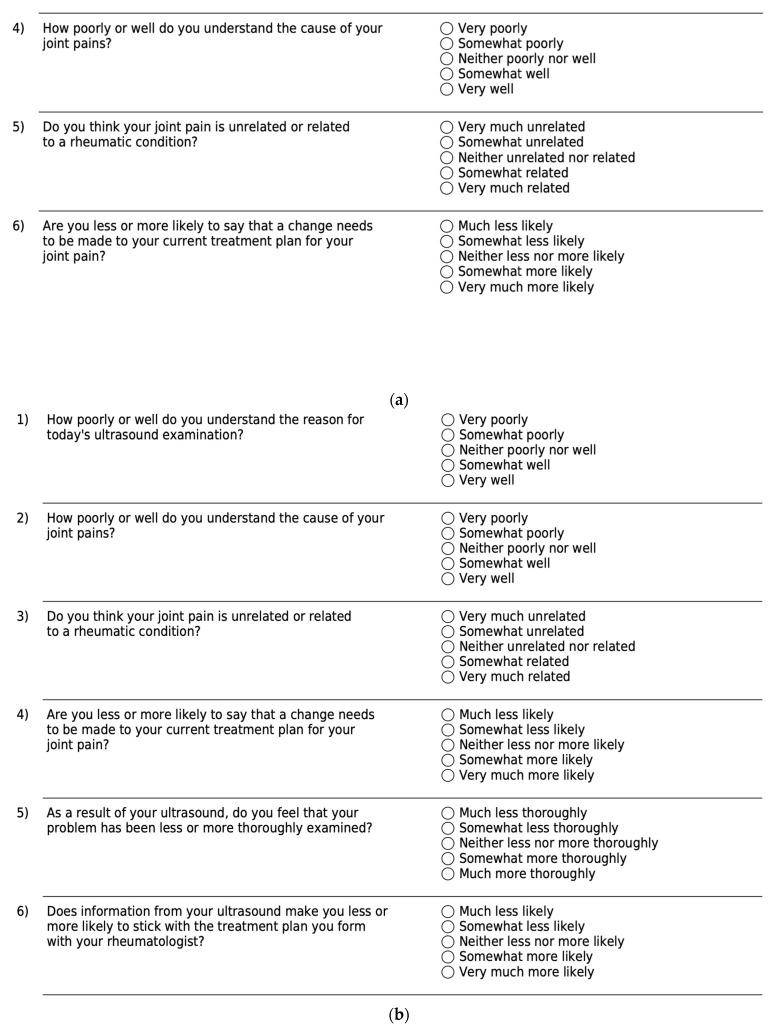
(**a**) Pre-visit surveys for patients. (**b**) Post-visit surveys for patients.

**Figure 27:**
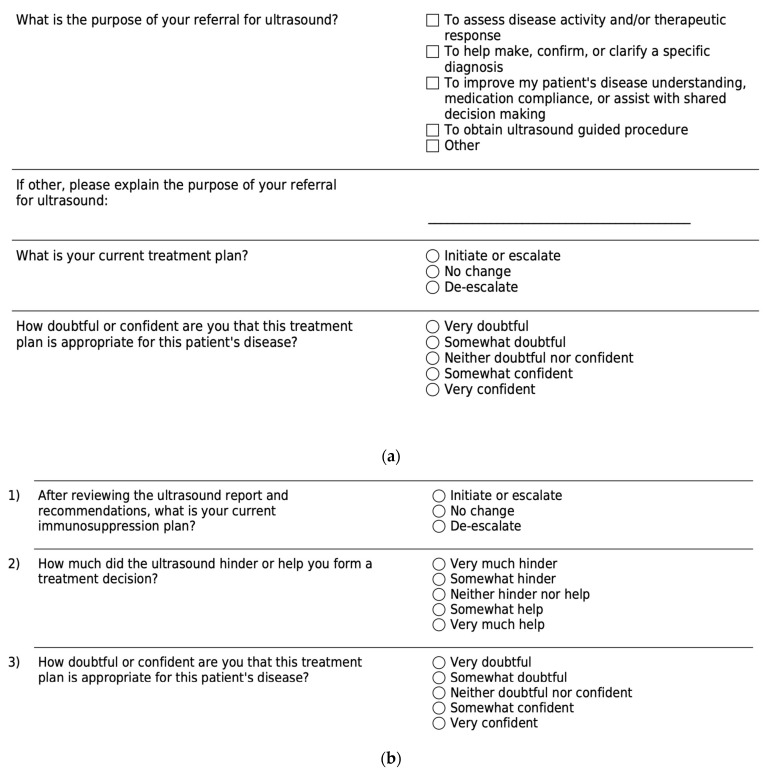
(**a**). Pre-visit surveys for providers. (**b**). Post-visit surveys for providers.

Methods: Eighteen randomly selected SSc patients with nonspecific hand pain were referred for musculoskeletal US exam. The study was conducted according to the guidelines of the Declaration of Helsinki and approved by the Institutional Review Board (or Ethics Committee) of Vanderbilt University Medical Center (protocol code #230657, Approved 05/18/2023). Informed consent was obtained from all subjects involved in the study. A pre- and post- US survey of both the patient (Figure 26a,b) and referring provider (Figure 27a,b) assessed diagnostic understanding and treatment changes.

**Figure 28:**
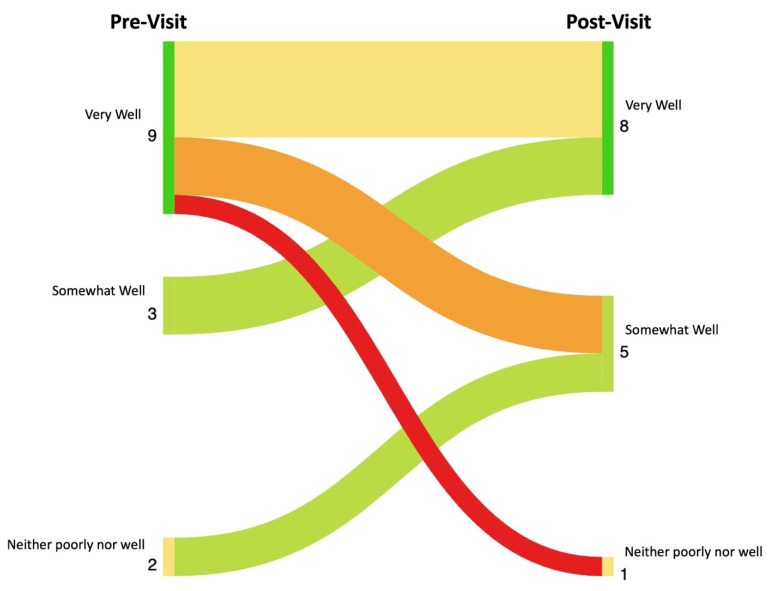
Sankey Plot showing changes between patients’ pre-visit and post-visit survey responses to the question “How poorly or well do you understand the cause of your joint pain?”. Color spectrum from bright green to dark red indicates degree of change in patient understanding. Bright green indicates significant improvement in understanding, bright red indicates significant worsening in understanding.

**Figure 29:**
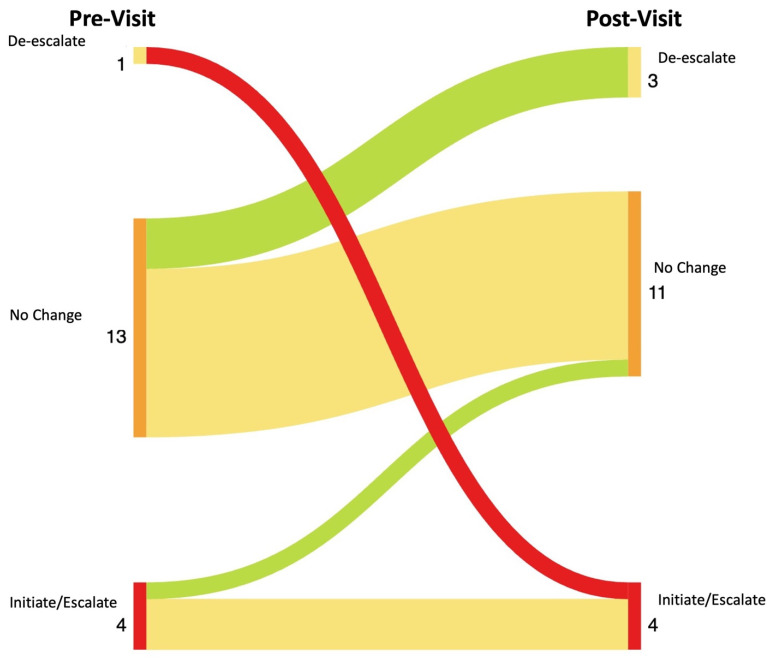
Sankey Plot showing changes between rheumatologists’ pre-visit and post-visit survey responses to the question “What is your current immunosuppression treatment plan?”. Color spectrum from bright green to dark red indicates degree of change in patient understanding. Bright green indicates significant improvement in understanding, bright red indicates significant worsening in understanding.

**Figure 30:**
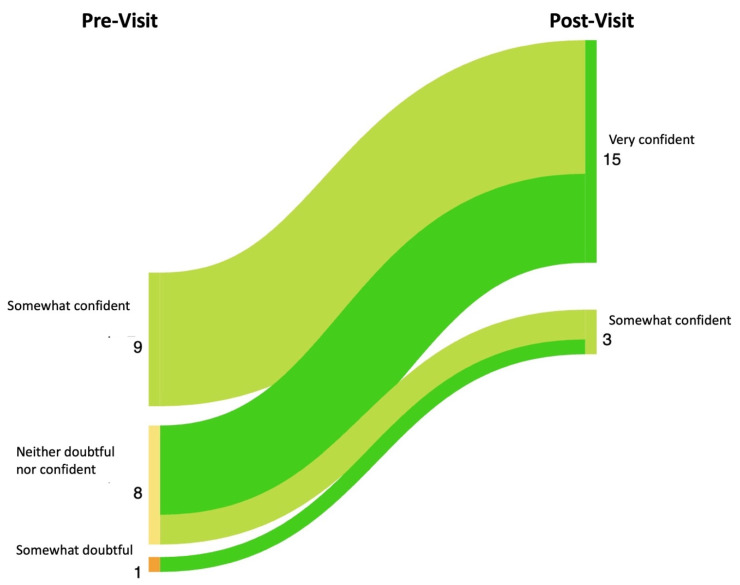
Sankey Plot showing changes between rheumatologists’ pre-visit and post-visit survey responses to the question “How doubtful or confident that this treatment plan (current immunosuppression plan) is appropriate for this patient’s disease?”. Color spectrum from bright green to dark red indicates degree of change in patient understanding. Bright green indicates significant improvement in understanding, bright red indicates significant worsening in understanding.

Results: The patients pre- and post-survey results are shown in Figure 28. In total, 14 of the 18 patients completed the survey. The providers’ pre- and post-survey results regarding their immunosuppression management plan are shown in Figure 29 and their confidence in their plan is shown in Figure 30.

After reviewing ultrasound findings, the referring providers’ confidence in treatment decision improved in all cases. All providers documented that the ultrasound “very much helped” form a treatment decision. The treatment plan was modified after US in 5 of 18 patients. US improved patients’ understanding of the cause of their joint pain in 5 of 14 patients. In total, 8 of the 14 patients rated that they understood the cause of their joint pain “very well” after their ultrasound exam. An amount of 13 of the 14 patients felt that their problem had been “somewhat more thoroughly” or “much more thoroughly” examined and felt that the information learned from their ultrasound made them “somewhat more” or “much more likely” to stick with the treatment plan formed with their rheumatologist. The data presented in this study are available on request from the corresponding author. The data are not publicly available due to privacy.

## 6. Conclusions

Relying on the physical exam and hand radiographs alone is inadequate for differentiating various etiologies of hand pain in SSc. We highlight a hand US protocol used at our center that has been shown to enhance provider confidence in treatment decisions and improve patient understanding of disease, potentially leading to improved treatment adherence. Standardized US assessment in SSc may provide a more comprehensive evaluation of hand pain and differentiate between various articular, periarticular, ischemic, skin, and nerve pathologies.

## Data Availability

Data supporting this manuscript is available upon reasonable request via email to corresponding author.
